# Mental Resilience, Mood, and Quality of Life in Young Adults with Self-Reported Impaired Wound Healing

**DOI:** 10.3390/ijerph19052542

**Published:** 2022-02-22

**Authors:** Jessica Balikji, Maarten M. Hoogbergen, Johan Garssen, Joris C. Verster

**Affiliations:** 1 Division of Pharmacology, Utrecht Institute for Pharmaceutical Sciences, Utrecht University, 3584 CG Utrecht, The Netherlands; jessicabalikji@gmail.com (J.B.); j.garssen@uu.nl (J.G.); 2 Division of Plastic Surgery, Catharina Ziekenhuis, Michelangelolaan 2, 5623 EJ Eindhoven, The Netherlands; dr.mmhoog@gmail.com; 3 Global Centre of Excellence Immunology, Nutricia Danone Research, 3584 CT Utrecht, The Netherlands; 4 Centre for Human Psychopharmacology, Swinburne University, Melbourne, VIC 3122, Australia

**Keywords:** impaired wound healing, slow-healing wounds, wound infection, mood, quality of life, sex differences

## Abstract

The purpose of this study was to evaluate the impact of self-reported impaired wound healing on quality of life, wellbeing, and mood. It was hypothesized that individuals with impaired wound healing report significantly poorer mood compared to healthy controls. An online survey was conducted among 2173 Dutch young adults (18–30 years old) to investigate mood, neuroticism, and mental resilience. Participants were allocated to a healthy control group (N = 1728) or impaired wound healing groups comprising a wound infection group (WI, N = 76), a slow-healing wounds group (SHW, N = 272), and a group that experienced both WI and SHW (the COMBI group, N = 97). The Kruskal–Wallis test was used to compare outcomes the groups. Compared to the healthy control group, the SHW and COMBI groups, but not the WI group, reported significantly poorer mood, increased neuroticism, reduced mental resilience, and reduced quality of life. An analysis evaluating sex differences found that negative effects on stress, mental resilience, and neuroticism were significantly more pronounced among women than among men. In conclusion, self-reported impaired wound healing is associated with poorer mood and reduced quality of life. To improve future wound care, these findings advocate for an interdisciplinary approach taking into account mood effects accompanying having impaired wound healing.

## 1. Introduction

Chronic wounds constitute an underestimated public health problem [1,2]. In the United States, approximately 8.2 million adults are diagnosed with chronic wounds with or without infection [3]. The financial cost of chronic wound treatments ranges from USD 28 billion to USD 31 billion [3]. Additionally, chronic wound patients frequently experience multiple disabling symptoms, including prolonged hospitalizations, disability, work loss, amputations in 25% of diabetic foot ulcer patients, and impaired quality of life [4].

Previous research reported multiple comorbidities, including anxiety and depression in patients with impaired wound healing [5,6,7,8]. Depression and anxiety both have a negative influence on quality of life, particularly when chronic diseases develop. Chronic wounds are wounds that fail to progress, or the response to treatment exceeds the normal expected healing time frame [9]. Impaired quality of life is observed, which is related to various aspects of wound healing, including physical symptoms caused by the wounds, complications due to underlying disease or treatment, and changes in functional capacity and mobility. These may result in dependency on others and socioeconomic consequences [1]. Patients with chronic wounds frequently report feelings of loss of self-control when the expected wound healing time is not met [10]. The impact of this loss of self-control may extend beyond the disease itself and negatively impact daily activities and cause concerns regarding patients’ future [10]. In addition, factors that have a high impact on a patient’s quality of life include frequent consultations and nursing appointments, loss of workdays, and sometimes losing their job [11].

In chronic wound patients, anxiety and depression have been associated with impaired wound healing, prolonged infection, and increased reoccurrence of wounds [6,8,12,13,14,15,16,17,18,19,20,21,22,23]. In a large European multicenter study that evaluated the psychological impact of skin diseases, the highest depression rate was reported by patients with leg ulcers [24]. Compared to the general population, depression is diagnosed three times more often in patients with impaired wound healing [8,25]. Approximately 30% of venous ulcer patients reported depression and/or anxiety [6], and this prevalence is about 40% in diabetic foot ulcer patients [23]. In diabetic patients, a bi-directional relationship has been found between having depression and chronic wounds. That is, diabetic patients with depression have an increased risk of developing chronic wounds and wound infection [26,27]. Diabetic patients with depression further showed a reduction in treatment compliance and poorer adherence to self-care responsibilities [28,29,30]. Treatment compliance and adequate self-care are essential for optimal wound healing [31]. Thus, an understanding of associated depressive behaviors, stress, and other mood changes can potentially improve treatment of chronic wound patients.

The literature reveals that some people have an increased susceptibility to experiencing mood changes compared to others, and there are also differences between people in regard to what extent they can cope with stress and mood fluctuations. This construct, so-called mental resilience, has increasingly become an important focus of research in the behavioral and medical sciences [32,33]. Mental resilience is defined as the ability to bounce back or recover from negative emotional experiences and the flexibility to adapt to the changing demands of stressful experiences. This construct includes different components, such as personal factors (coping mechanism), family, and social protective factors [34]. A positive coping mechanism results in higher levels of confidence and self-esteem, and quality of life. Mental resilience leads to protection of an individual against the impact of traumatic events [35,36], but it is also a tool that can be used to recover from these events [33,37,38,39]. Hence, it has been suggested that mental resilience is a form of behavioral immunization [40,41]. Thus, experiencing a stressful episode strengthens an individual’s resistance to future stressful events. Research has shown a significant association between resilience and psychological health though different coping strategies, including affective, cognitive, and behavioral processes [42]. Furthermore, resilience as an important psychological resource is positively correlated with extraversion [43], is negatively correlated with neuroticism [44], and is associated with increases in positive affect and decreases in negative affect [45,46]. Resilience plays an essential role in improving well-being and general life satisfaction, especially for the college students [47]. Other studies found that happiness was the most significant predictor of self-rated health [48].

Although impaired wound healing is most frequently observed in older individuals [49], it is also seen in younger age groups [50]. However, scientific research on younger age groups with impaired wound healing is scarce. Therefore, the aim of this study was to compare mood in young adults with and without self-reported impaired wound healing. It was hypothesized that individuals with impaired wound healing report significantly poorer mood compared to healthy controls.

## 2. Materials and Methods

Dutch university students (18 to 30 years old) were invited via Facebook to participate in a study on food and health. An online survey could be completed via www.surveymonkey.com (accessed on 3 February 2022). The study was approved by the University of Groningen Psychology Ethics Committee (Approval code: 16072-O, date of approval: 25 October 2016), and all participants provided online informed consent before starting the survey.

### 2.1. Participants

Participants indicated whether or not they had experienced wound infections or slow-healing wounds during the past year. They were then allocated to one of the following four groups: (1) a control group without impaired wound healing, (2) a wound infection (WI) group, (3) a slow-healing wounds (SHW) group, or (4) the COMBI group (both WI and SHW).

### 2.2. Mood

The tension, depression, and anger scales of the short version of the Profiles of Mood States (POMS-SF) were completed [51,52]. Items were scored on a 5-point Likert scale, with answering possibilities ranging from 0 (not at all) to 4 (extremely). The sum scores for the three scales were calculated. Cronbach’s alpha of the tension, depression, and anger scales were 0.80, 0.90, and 0.89, respectively [52]. In addition, the Depression Anxiety Stress Scales (DASS-21) questionnaire was completed [53]. The 21 items were scored on a 4-point Likert scale, with answering possibilities ranging from 0 (not at all) to 3 (very much or most of the time). The sum scores were computed for the three scales. A higher scale score implies a higher level of depression, anxiety, or stress. In previous research with students in the Netherlands, the depression, anxiety, and stress scales had a Cronbach’s α of 0.91, 0.86, and 0.85, respectively [54].

### 2.3. Neuroticism

The 12-item neuroticism scale of the Eysenck Personality Questionnaire—revised Short Scale (EPQ-RSS) was completed [55,56]. Items could be answered with ”yes” or ”no”. Sum scores range from 0 to 12, with higher scores implying more neuroticism. A previous study in Dutch students found a Cronbach’s α of 0.81 for the neuroticism scale [56].

### 2.4. Mental Resilience

Mental resilience was evaluated with the Brief Resilience Scale [57]. The 6-item BRS assesses one’s ability to recover from stress, i.e., the ability to bounce back. The items are answered on a 5-point Likert scale, ranging from “strongly disagree” to “strongly agree”. Scores range from 1 to 5, and the mean score of the six items was calculated, with higher scores implying higher levels of mental resilience. Previous studies showed that the level of mental resilience correlated significantly with personality, coping strategies, and health correlates [57]. Cronbach’s α of the BRS ranged from 0.80 to 0.91 [57].

### 2.5. Quality of Life

Quality of life was measured with the 5-item World Health Organization (WHO-5) Well-Being Index [58]. Items are answered on a 5-point Likert scales, ranging from 0 (“At no time”) to 5 (“All of the time”). The sum score of the items, multiplied by 4, is the outcome measure of the WHO-5, with higher scores corresponding to a higher level of wellbeing. Previous studies revealed that the WHO-5 outcome significantly correlated with psychological constructs such as mental resilience and self-esteem, and mental health outcomes such as depression [58,59]. Cronbach’s alphas of 0.85 to 0.91 have been reported for the WHO-5 [60,61].

### 2.6. Statistical Analyses

Statistical analyses were conducted with SPSS (IBM Corp. Released 2013. IBM SPSS Statistics for Windows, Version 28.0. Armonk, NY, USA: IBM Corp.). Mean and standard deviation (SD) were computed for each variable, and the data distribution of each variable was checked for normality with the Kolmogorov–Smirnov test and by visual inspection. The outcome of these analyses revealed that the data were not normally distributed. Hence, nonparametric tests were conducted to further analyze the data. The comparisons between the groups (control, SHW, WI, and COMBI group) were performed with the Kruskal–Wallis test. To account for multiple comparisons, Bonferroni’s correction was applied (*p* < 0.0083 for significance).

## 3. Results

Data from N = 2173 participants (83.8% women) were used for the analysis. The demographics of the participants are summarized in Table 1. No significant differences were found between the groups. Psychological correlates are summarized in Table 2.

Compared to the control group, both stress and anxiety scores on the DASS21 were significantly higher for the SHW group (both *p* < 0.001) and the COMBI group (both *p* < 0.001). DASS21-Depression scores of the SHW group (*p* < 0.001) and the COMBI group (*p* < 0.001) were also significantly higher than those of the control group. Depression assessed with the POMS-SF-Depression scale was consistent with these findings. Relative to the control group, POMS-SF-Depression scores were significantly higher for the SHW group (*p* < 0.001) and the COMBI group (*p* < 0.001), and the difference between the WI and COMBI group was also statistically significant (*p* = 0.004). POMS-SF-Tension scores of the SHW group (*p* < 0.001) and the COMBI group (*p* < 0.001) were significantly higher than those of the control group. Finally, POMS-SF-Anger scores of the SHW group (*p* < 0.001) and the COMBI group (*p* = 0.001) were significantly higher than those of the control group. 

Neuroticism scores of the SHW group (*p* < 0.001) and COMBI group (*p* < 0.001) were significantly higher than those of the control group. The COMBI group had also significantly higher scores than those of both the WI groups (*p* < 0.001). Compared to the control group, mental resilience ratings were significantly lower for the SHW group (*p* = 0.002) and the COMBI group (*p* = 0.002). Finally, compared to the control group, quality of life was rated significantly poorer by both the SHW group (*p* < 0.001) and the COMBI group (*p* < 0.001).

### Sex Differences

Since the number of included males in the impaired wound healing groups was too small to allow well-powered analyses, we combined the groups into one impaired wound healing group to evaluate potential sex differences. The results are summarized in Table 3. In the control group, women scored significantly higher than men on POMS-SF-Tension and neuroticism and significantly lower than men on mental resilience (all *p* < 0.001), and they reported a significantly poorer quality of life than men (*p* < 0.001). In the impaired wound healing group, mood scores were also generally higher in women than in men, and statistically significant sex differences were found for DASS21—Stress (*p* = 0.004), mental resilience (*p* < 0.001), and neuroticism (*p* < 0.001). For men, no significant differences were found between the control group and impaired wound healing group. Compared to the women of the control group, women of the impaired wound healing group scored significantly poorer on all assessments (all comparisons *p* < 0.001).

## 4. Discussion

The current study showed significant relations between impaired wound healing and negative mood, reduced mental resilience, and poorer quality of life. The findings confirm previous research, showing that negative mood changes related to impaired wound healing are a chronic burden for affected patients [2]. Previous studies have shown the contribution of the skin’s microbiome to host homeostasis, allostasis, and the pathogenesis of disease. Complex immune mechanisms connect the skin’s microbiome with other organs, including the brain [62], and research has shown that this relationship is bidirectional [63]. That is, alterations in skin integrity may lead to significant modification of patients’ psychological health [64,65].

Maladaptive ways to cope with stress (e.g., rumination) may result in prolonged depressive episodes, whereas active approaches (e.g., regular exercise) may be a protective factor that reduces stress. When facing adversities, females also have a different coping style with stressors. That is, whereas males usually employ active strategies to cope with stress, females more often tend to ruminate over problems. In the current study, the negative effects on stress, mental resilience, neuroticism, and quality of life were significantly greater among women than among men. Our findings are in agreement with other research showing that sex moderated the interplay between resilience and mental health [66,67,68,69]. Additionally, females often report lower levels of self-confidence and self-efficacy, and less personal and material resources than males. Together, this may result in lower mental resilience among females [68]. Compared to males, females may experience more happiness from contact with family and their social network, and, therefore, they may be more sensitive to stressors that interfere with these relationships [70,71]. Stress has been reported to be the most important predictor of mental health status of females [72,73], and traditional sex roles in upbringing may contribute to later-life sex differences in coping [70,71]. 

When interpreting the data of this study, several limitations should be taken into account. Firstly, the assessment in this study was self-reported and retrospective. Therefore, the responses by participants may be influenced by personal perceptions, and recall bias may also have had an impact on these responses. Secondly, participants were allocated to the impaired wound healing groups or the control group based on self-report. There was no clinical diagnosis to support this classification, and the classification was simply based on reporting the presence or absence of slow-healing wounds and/or wound infection over the past year. It is unknown how participants evaluated whether their wound healing should be regarded as impaired/slow, but it could be speculated that they compare their wound healing to that of peers, information from the internet, or their personal situation before acquiring a disease that is characterized by slow wound healing (e.g., diabetes). Another consequence of our approach is that the type and severity of wounds was not assessed. Moreover, possible causes of the wounds or infection (e.g., underlying disease such as diabetes, surgery, or an accident) were not recorded. Future studies should collect these data and confirm wound healing status by diagnosis made by a clinician. It can then also be evaluate to what extent these factors influence the impact of impaired wound healing on mood and quality of life. Thirdly, while mental health problems were evaluated by DASS-21 and EPQ-RSS, it should be noted that these scales were developed for screening purposes and not to diagnose patients. These instruments offer severity scores on related symptoms of depression, anxiety, stress, and neuroticism, but these scales do not have cut-off values for screening positive or negative for a diagnosis. A formal diagnosis requires a thorough examination by a trained psychologist or physician. In future research, it would also be interesting to evaluate the possible influence of different mental conditions on the judgment of wound healing. There is a possibility of a reverse relationship in that a psychiatric disorder could make healing feel delayed, while this objectively is not the case. The latter is an important topic for future research. In this study, we simply asked the participants if they experienced slow wound healing or wound infection in the past. In future, prospective studies, the wound healing process should be followed in real time. Assessments by a clinician could then be complemented with self-assessments of the wound healing, such as the Pictorial Representation of Illness and Self Measure (PRISM) [74,75], and allow a direct comparison between clinician and patient assessments. Fourthly, the sample consisted of young adults (18 to 30 years old). It is unknown to what extent the results are representative of older age groups. Moreover, females were overrepresented in our sample. However, this reflects the common sex distribution at Dutch universities. The sample size was sufficient to evaluate potential sex differences between the impaired wound healing group and the control group but too small to differentiate between the different wound healing groups. Therefore, the impaired wound healing groups were combined for the statistical analysis. Having a large control group and relatively small impaired wound healing groups reflects the relative low occurrence of impaired wound healing in the general population. Having a large control group is an advantage of the study, as this increases assurance that the obtained mood ratings of the control group more closely correspond to the actual mood ratings of the entire population of young adults. However, in future prospective studies, the sample sizes of the impaired wound healing groups and control group could be equalized by matching cases and controls. Finally, lifestyle factors (e.g., nutrition and physical activity) and social support [76] may have a relevant impact on wound healing, but these were not assessed.

Notwithstanding these limitations, the analysis revealed significant associations between impaired wound healing and negative mood. These associations are in line with the literature suggesting a more general bi-directional relationship between having non-communicable diseases and mood and wellbeing [77]. For example, depressed mood has also been reported for patients with congenital heart disease or diabetes [78,79]. The current findings justify further research on psychosocial factors associated with wound healing, preferably combining both subjective and objective assessments in patients with confirmed impaired wound healing, mood disorders, or stress. An interdisciplinary approach taking into account mood effects accompanying having impaired wound healing could improve future wound care.

## 5. Conclusions

Self-reported impaired wound healing is associated with poorer mood and reduced quality of life. To improve future wound care, these findings advocate for an interdisciplinary approach taking into account mood effects accompanying having impaired wound healing.

## Figures and Tables

**Table 1:** Demographics.

Demographics	Control Group	WI Group	SHW Group	COMBI Group
N	1728	76	272	97
Sex (m/f)	287/1441	13/63	45/227	8/89
Age	21.3 (2.1)	21.3 (2.0)	21.2 (2.1)	21.0 (2.0)

Abbreviations: SHW = slow-healing wounds, WI = wound infection, COMBI = slow-healing wounds and wound infection.

**Table 2:** Psychological correlates of wound infection and slow-healing wounds.

Psychological Correlates	Control Group	WI Group	SHW Group	COMBI Group
POMS-SF—Tension	3.9 (4.3)	4.4 (4.2)	5.4 (5.2) *	6.6 (5.3) *
POMS-SF—Depression	3.1 (4.8)	4.0 (5.7)	5.5 (6.8) *	6.2 (6.0) * γ
POMS-SF—Anger	2.2 (3.2)	2.7 (4.2)	3.5 (4.4) *	3.3 (3.6) *
DASS21—Anxiety	7.0 (7.1)	8.3 (6.5)	9.5 (8.7) *	11.8 (9.6) *
DASS21—Depression	5.1 (6.4)	5.8 (6.5)	7.9 (8.3) *	9.5 (8.8) *
DASS21—Stress	9.1 (7.4)	11.1 (7.3)	12.5 (8.4) *	14.3 (8.8) *
Mental resilience	3.3 (0.7)	3.3 (0.7)	3.1 (0.7) *	3.0 (0.7) *
Neuroticism	8.3 (5.3)	9.1 (5.9)	10.6 (5.6) *	12.5 (5.7) * γ
Quality of Life	53.4 (15.9)	50.8 (14.1)	47.7 (15.6) *	46.3 (13.7) *

Significant comparisons with the control group (*p* < 0.0083, applying Bonferroni’s correction for multiple comparisons) are indicated by *, significant differences between the WI and COMBI groups are indicated by γ. Abbreviations: SHW = slow-healing wounds, WI = wound infection, COMBI = slow-healing wounds and wound infection, POMS-SF = profile of mood states—short form, DASS21 = depression anxiety stress scale 21-items.

**Table 3:** Psychological correlates of impaired wound healing according to sex.

	Control Group		Impaired Wound Healing Group	
Psychological Correlates	Men	Women	Men	Women
POMS-SF—Tension	3.0 (3.4)	4.1 (4.4) ^†^	4.2 (4.3)	5.7 (5.1) *
POMS-SF—Depression	2.4 (4.0)	3.2 (5.0)	3.8 (4.9)	5.6 (6.6) *
POMS-SF—Anger	2.4 (3.7)	2.1 (3.2)	3.4 (4.9)	3.3 (4.1) *
DASS21—Anxiety	6.1 (6.3)	7.1 (7.3)	7.5 (6.8)	10.1 (8.8) *
DASS21—Depression	4.8 (6.3)	5.1 (6.4)	6.0 (7.0)	8.2 (8.4) *
DASS21—Stress	7.1 (6.9)	9.5 (7.5) ^†^	9.5 (7.7)	13.2 (8.4) * ^†^
Mental resilience	3.7 (0.7)	3.2 (0.7) ^†^	3.5 (0.7)	3.1 (0.7) * ^†^
Neuroticism	5.9 (4.7)	8.8 (5.3) ^†^	7.8 (6.1)	11.2 (5.6) * ^†^
Quality of Life	56.4 (15.9)	52.8 (15.9) ^†^	51.8 (16.4)	47.2 (14.6) *

Significant differences between the control group and impaired wound healing group (*p* < 0.005, applying Bonferroni’s correction for multiple comparisons) are indicated by *. Significant sex differences (*p* < 0.005, applying Bonferroni’s correction for multiple comparisons) are indicated by ^†^.

## Data Availability

The data are available upon reasonable request from the corresponding author.
